# Googling Location for Operating Base of Mobile Stroke Unit in Metropolitan Sydney

**DOI:** 10.3389/fneur.2019.00810

**Published:** 2019-08-06

**Authors:** Thanh G. Phan, Richard Beare, Velandai Srikanth, Henry Ma

**Affiliations:** ^1^Stroke Unit, Monash Health, Melbourne, VIC, Australia; ^2^Stroke and Aging Research Group, Medicine, School of Clinical Sciences, Monash University, Melbourne, VIC, Australia; ^3^Department of Medicine, Frankston Hospital, Peninsula Health, Melbourne, VIC, Australia; ^4^Central Clinical School, Monash University, Melbourne, VIC, Australia; ^5^Developmental Imaging, Murdoch Children Research Institute, Melbourne, VIC, Australia

**Keywords:** stroke, mobile stroke unit, optimization, google maps, endovascular clot retrieval

## Abstract

**Background and purpose:** The recent advances in stroke therapy have placed focus on delivering care within the first hour after stroke onset (golden hour), principally through the use of Mobile Stroke Unit (MSU) to bring the hospital to the patient. The aim of this project is to search the location of MSU hub in Sydney, Australia, optimizing for catchment, transport to nearest thrombolysis and endovascular clot retrieval (ECR)/thrombectomy capable hospital and population at risk.

**Methods:** Traveling time was performed using *ggmap* package in R to interface with Google Maps application program interface (API). This analysis estimates the travel time from the centroids of each suburbs to five potential MSU hubs (Royal Prince Alfred, Prince of Wales, Royal North Shore, Liverpool, and Westmead hospitals) and eight thrombolysis capable hospitals. It is proposed that the MSU should be deployed at ECR hub to cover the suburbs, not well-covered by thrombolysis and ECR capable hospitals. This step was performed by assigning membership to hospitals within 30 min traveling time to the ECR hub. The base hub of the MSU was proposed as the closest hub (providing ECR) to the least well-served suburbs. The population serviceable by MSU was estimated using stroke incidence studies in Melbourne and Adelaide.

**Results:** The largest population, serviceable by MSU within 30 min (4,606 cases), 45 min radius (8,918 cases), and 60 min (10,084 cases), was Royal North Shore followed by Royal Prince Alfred, Liverpool, Westmead, and Prince of Wales hospitals. Prince of Wales hospital has the smallest catchment within 30 min (3,078 cases), 45 min (7,721 cases), and 60 min (9,984 cases). Suburbs at the edge of metropolitan Sydney such as the Northern Suburbs are less well-served by thrombolysis and ECR capable hospitals. There are 10 suburbs within 30 min travel of one hospital. The remainders are within 30 min of two or more hospitals.

**Conclusions:** Any of the five endovascular clot retrieval capable hospitals are capable of serving as a hub for MSU. We provide a method to identify the hub based on location of suburbs less well-served by other hospital.

## Introduction

The recent advances in stroke therapy have placed focus on delivering care within the first hour after stroke onset (golden hour) (1). A potential solution is the use of Mobile Stroke Unit (MSU) to bring the hospital to the patient for providing thrombolysis. Since the publications of the endovascular clot retrieval hospital (ECR)/thrombectomy trials in 2015 (2–7), a new indication for MSU is to triage transportation of patients to the appropriate hospital for ECR. The first randomized trial in 2012 showed an absolute difference of 16 min (8); this had occurred in the context of the MSU operating only a short range from base (8). Subsequent publications showed a difference of 39 min in time to thrombolysis (9). The MSU is a modified ambulance, which sometimes approaches the size of a truck. It is equipped with CT scanner and a mobile pathology laboratory and staffed by ambulance officer, nurse, radiographer, and in some places, doctor. There is strong interest in developing this platform and a model currently exist in Melbourne, Australia's second largest city.

There are several models for operating MSU around the world (1). In one model, MSU is located in a central location in a fire station and travel up to 16 min from base (10). A health economic analysis in Germany suggested MSU can provide service up to 30 km radius from base (11). The model in Edmonton, Canada provides service in rural Alberta up to a radius of 250 km^2^ (12). The varying operating radii of MSU suggest that its deployment has not been formally evaluated. In the Australian context, the Melbourne model operates MSU from a centrally located major hospital, Royal Melbourne Hospital, and travel a 20 km radius from 8 a.m. to 6 p.m. (13). This model had occurred at Royal Melbourne Hospital and was nominated as the first designated ECR hub in Melbourne (14). Subsequently, a geospatial optimization analysis suggested that such a model can operate up to 76 min from base (15). In another paper, in this special issue of Frontiers in Neurology, the role of MSU in performing triaging of patients for therapy in the rural setting was explored (16). In this study, we propose the use of *ggmap* interface to Google Maps API to assign the base for operating MSU in Sydney based on proximity of the hub to the suburbs less well-resourced in terms of closeness to hospitals providing ECR and thrombolysis (14). The optimization will be performed for catchment, transport to nearest thrombolysis-capable hospital, and population at risk of stroke. Sydney is the ideal site for this study, as the locations of the ECR hubs have not been finalized and all potential ECR hubs are eligible as the base (17).

## Methods

### Setting

Sydney is the capital city of the state of New South Wales, Australia with a population of ~5.1 million. The number of strokes has been estimated using the 2016 census data (1) for each age band and the stroke incidence study in Melbourne and Adelaide (18, 19). This study involves simulations (no patient data are used) and as such received a waiver from the Monash Health Human Research Ethics Committee.

### ECR Capable Hospitals in Sydney

At present there is no official statewide protocol for ECR in Sydney (20). There are five hospitals (Royal Prince Alfred, Prince of Wales, Royal North Shore, Liverpool, and Westmead) capable of acting as ECR hubs. There is one ECR hub, located 159 km north of Sydney. Similar to the Melbourne model of basing the hub in a major teaching hospital, these hubs may serve as the base for MSU. In addition, there are eight hospitals providing thrombolysis service in metropolitan Sydney and eight outside of metropolitan Sydney (20).

### Google Maps API

Estimation of ambulance travel times and potential hospital catchment were performed using the *ggmap* (21) (R Project for Statistical Computing, version 3.4.4) interface to the Google Maps Distance Matrix API. The transport time, from each hospital to each suburb centroid, was computed during peak morning traffic. Interactive web-based maps of the models were generated using *leaflet* (R package) with tiles from OpenStreetMap (OpenStreetMap contributors; for copyright, see www.openstreetmap.org/copyright) (22).

### Optimization

We formulated the problem as a variant of the maximum coverage (23). In a traditional maximum coverage problem, the hospital sites are not yet determined and the optimization is performed to allocate the sites, which provide best coverage for the area. In this case, the hospitals have been built and each have their own capabilities such as thrombolysis capable or ECR capable. We propose that the MSU should be deployed to cover the suburbs, not well-covered by thrombolysis and ECR capable hospitals. This step was performed by assigning membership to hospitals within 30 min (24). From an equitable point, the suburbs, which have least memberships, were considered to be more likely to benefit from MSU compared to suburbs with multiple memberships (25). The base hub of the MSU was proposed as the closest hub to the least well-served suburbs.

In addition, we provide a sensitivity analysis by estimating the population at risk of stroke serviced by MSU. This analysis was done by extending the base from 30 to 60 min (24).

## Results

**Figure 1:**
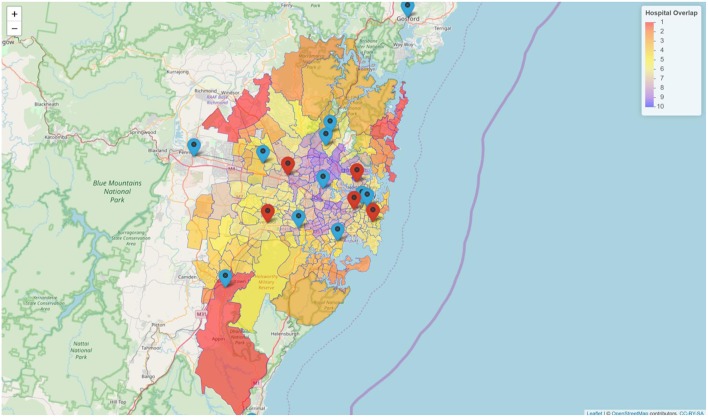
Coverage of suburbs by thrombolysis and clot retrieval hospitals. There are five ECR capable hospitals (red icons) and eight thrombolysis capable hospitals (blue icon) in metropolitan Sydney. The red color polygon indicates less overlap and the purple color indicates high number of overlap.

**Figure 2:**
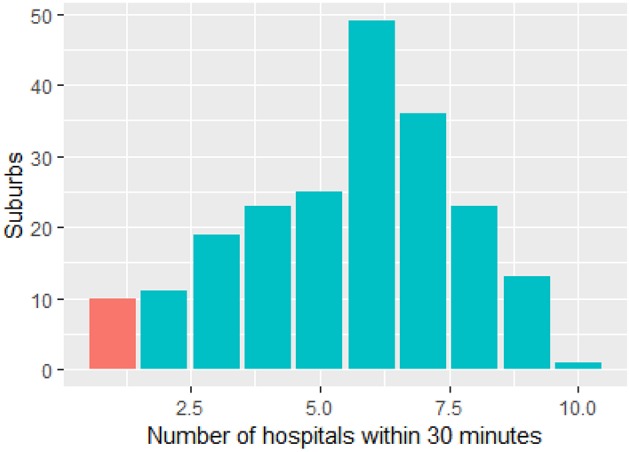
Relationship between suburbs and hospital coverage in Sydney. There are 10 suburbs within 30 min travel of one hospital (red). The remainders are within 30 min of two or more hospitals.

**Figure 3:**
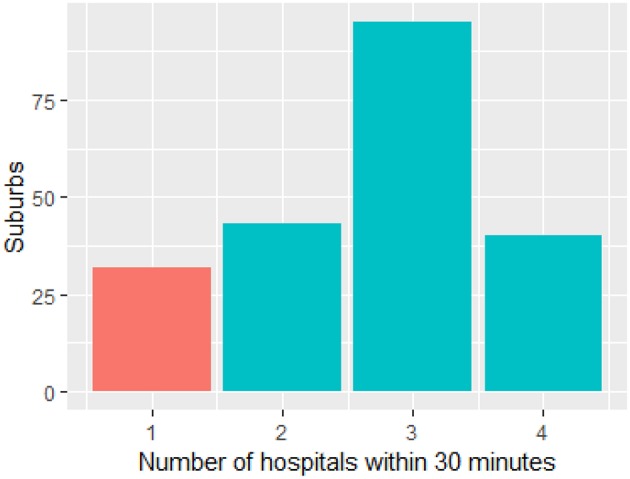
Relationship between number of suburbs and coverage by ECR capable hospitals. Thirty-two suburbs are within 30 min of one ECR capable hospital (red). ECR, endovascular clot retrieval.

The map (Figure 1) illustrates the suburbs, well-served by overlapping catchment of other hospitals. These are suburbs, located between the following Royal Prince Alfred, Westmead, Sydney Adventist, Bankstown, and St. George. Concord hospital is located at the center of these suburbs with overlapping catchment. By contrast, the suburbs at the edge of metropolitan Sydney such as the Northern Suburbs are less well-served by thrombolysis and ECR capable hospitals. On the map these Northern suburbs have values of one only, indicating that they are serviced by only 1 hospital. Figure 2 is a histogram of the suburbs and their 30 min proximity to a thrombolysis and ECR capable hospitals. Ten suburbs are within reach of one hospital within 30 min and 200 suburbs are within reach of two or more hospitals within 30 min. Thirty suburbs are within 30 min of one ECR capable hospital. One hundred and thirty-five suburbs are within 30 min of more than three ECR capable hospitals (Figure 3).

The largest population, serviceable by MSU within 30 min (4,606 cases), 45 min radius (8,918 cases), and 60 min (10,084 cases), was by Royal North Shore hospital followed by Royal Prince Alfred, Liverpool, Westmead, and Prince of Wales hospitals (Table 1). Prince of Wales hospital has the smallest catchment within 30 min (3,078 cases), 45 min (7,721 cases), and 60 min (9,984 cases). Within 60 min from hub, five sites differ by 140 cases. Three of the sites have two associated spokes hospitals (Table 2; Figure 1).

**Table 1:** Projected number of patients with stroke serviced by different combinations of ECR hubs.

**Hospital**	Melbourne incidence ^*^30 min	Melbourne incidence ^*^45 min	Melbourne incidence ^*^60 min	Melbourne incidence ^*^20 km	Melbourne incidence ^*^30 km	Melbourne incidence ^*^50 km	**Adelaide incidence ^#^30 min**	**Adelaide incidence ^#^45 min**	**Adelaide incidence ^#^60 min**	**Adelaide incidence ^#^20 km**	**Adelaide incidence ^#^30 km**	**Adelaide incidence ^#^50 km**
RPA	4,247	8,475	10,047	4,717	7,541	9,944	2,465	4,911	5,824	2,736	4,367	5,762
LPH	3,772	8,475	10,047	2,986	6,121	9,143	2,198	4,337	5,519	1,738	3,558	5,306
WH	4,294	7,988	9,844	3,550	6,588	9,859	2,498	4,640	5,708	2,064	3,827	5,713
POW	3,078	7,721	9,984	2,874	5,836	9,424	1,784	4,468	5,788	1,666	3,376	5,453
RNS	4,606	8,918	10,084	3,650	6,497	9,349	2,665	5,164	5,845	2,117	3,759	5,410

* *Melbourne stroke incident study*.

# *Adelaide stroke incident study. ECR, endovascular clot retrieval; RPA, Royal Prince Alfred; WH, Westmead Hospital; POW, Prince of Wales Hospital; RNS, Royal North Shore Hospital*.

**Table 2:** Thrombolysis capable spoke hospital proximity to ECR hub.

**Hospital**	**Spoke hospitals 1 (min)**	**Spoke hospitals 1 (km)**	**Spoke hospital 2 (min)**	**Spoke hospital 2 (km)**
RPA	25.2	12.9		
LPH	17.1	10.7	26.0	28.5
WH	20.7	12.7	33.0	29.3
POW	24.4	16.1		
RNS	25.8	19.1	22.7^*^	16.4^*^

* *Privately operated hospital. ECR, endovascular clot retrieval; RPA, Royal Prince Alfred; WH, Westmead Hospital; POW, Prince of Wales Hospital; RNS, Royal North Shore Hospital*.

## Discussion

In this study, we have used data driven method to map location of MSU base in Sydney using Google Maps API estimate of travel time from the ECR and thrombolysis capable hospitals. This issue arises as there are five ECR capable hospitals in Sydney; each has their merits of operating as a base for MSU. Using the idea of equity, an ECR hub was identified as the one that was closest to the suburbs, least served by ECR and thrombolysis capable hospitals. Our methodological approach is of use when planning to invest in MSU.

The use of geographical information systems for health service design is evolving since 2017 (14, 15, 26–30). In this study, we have accessed Google Maps API for traffic data due to our familiarity with this platform (14, 15, 26–30). Use of Google Maps API for this purpose has been done in Australia and North America (14, 15). Those planning similar studies in other part of world such as Republic of Korea (South Korea) would need to access to Daum, Naver, or VWorld Map API. In People's Republic of China, Baidu Map API is preferred to Google Maps API. These tools can be combined with *ggmap* or other tools discussed in this special issue of *Frontiers in Neurology (Stroke)*. In the Republic of China (Taiwan), Google Maps API is available for travel time estimation.

The approach we have taken for evaluating MSU base in Sydney is different from that in Melbourne (15). The MSU base in Melbourne has already been chosen and stroke neurologists and nurses from Royal Melbourne Hospital form part of the crew for the MSU (30). The objective of the paper is to ascertain the range of the MSU in Melbourne as the original intention was to restrict its operation to just 20 km from base. Our approach shows that in Melbourne, MSU can operate as far as 76 min from base and would provide superior time with respect to administration of TPA (31). With the range of operation in mind, it is proposed that provision of MSU service to an area, well-served by multiple adjacent hospitals, would not be equitable. The alternative argument is that MSU provides a mean to reduce door-in to door-out time, even within these well-covered suburbs. The argument would be that even among suburbs, well-served by other hospitals, there is still the issue of door-in to door-out time, which can vary from 85 min in Rhode Island (32) to 106 min in Melbourne (31).

It is possible that the issue of deciding between thrombolysis and ECR capable hospitals and MSU in a well-service catchment has not been explored in detail, as some of the centers operating MSU have only one ECR hub and have a small catchment population. Sites such as Lucas County serves a population of 433,689 (33); New Jersey model serves a modest population of 460,000 (34); and the Cleveland model serves a population of 390,000 (35). In Germany, there is exploration on the deployment of MSU in rural areas (16). In Edmonton, Canada, there is extension of the operating radius as far as 250 km from base (12).

Similar to the Houston and Melbourne model of basing the MSU hub in a major public teaching hospital (36), the rationale for the use of major hospital is that a stroke neurologist or neurology trainee from that hospital participates in the team. This has been the case for the first 2 years in Houston (36). This approach is different from the original model in Berlin in which the base is in a centrally located fire station in Berlin. If the desire is to locate the base in a central location, then the choice would be Royal Prince Alfred hospital, which is closest to the center of Sydney. However, such a model would be less equitable, given the number of suburbs, serviced by other hospitals. A counter argument would be that the suburbs with service by multiple hospitals is that where people live with risk of stroke.

Projection of patients at risk of stroke is based on stroke incidence studies from Melbourne (on the East coast of Australia) and Adelaide (on the South Coast of Australia) (18, 19). The studies were performed 13 years apart with the latter study from Adelaide documenting a much lower stroke incidence. The improved stroke incidence can be due to different methodology or improved stroke prevention strategy. As such we have provided data on the estimated number of stroke cases using stroke incidence both studies.

A limitation of this study is that it is based on simulations and not observed cases of stroke. We had circumvented this by simulating stroke cases in the centroid of each suburb. This approach provides an average of the trip time. Another limitation in the proposal for MSU is the issue of cost effectiveness, which has not yet been done. Current studies are underway on cost effectiveness of analyses of MSU (37, 38). A critical issue for cost effectiveness is the requirement to demonstrate difference in primary outcome on disability; so far the trial confirmed reduction in time to give thrombolysis only (9). The findings with reduction in time to thrombolysis are consistent whether in Europe or North America (35). However, this study has not focused on clinical outcome. The flow chart from this study shows another issue while constructing the cost effectiveness analysis (35). Twenty eight of 100 patients, treated by MSU, did not have stroke and 30 were classified as possible acute ischemic stroke. A further 217 trips (out of total 317 trips) were canceled prior to MSU arrival in this Cleveland Clinic study (35).

Depending on the configuration of MSU, the cost has been estimated to be around US$750,000 to US$1,400,000. The running cost of operating MSU during office hour is estimated to be around US$1,000,000 (34). Recently, a group in Lucas County Ohio reported on their initial experience with operating MSU 24 h a day (33). Data on 24-h operation and cost effectiveness study such as these will help inform the need for MSU.

This study was not designed to address the issue of cost effectiveness or propose the purchase of MSU but rather to evaluate the location for deployment of MSU. In the process of performing these analyses, there were issues on clustering or proximity of hospitals with thrombolysis and ECR capability. There may be a need to allocate resources equitably from inner Sydney to outer Sydney while planning the location of MSU hub. This is an issue that is not unique to Australia but is also relevant in Europe (16) and Canada (12). In London, this had been addressed by reducing the number of hospitals providing thrombolysis service (24, 39). The model was designed in such a way that no Londoner should be more than 30 min away from a hyperacute stroke service (24). A recent analysis on the effect of centralizing and rationalizing of stroke service in two metropolitan cities in England shows that the changes result in improved outcome (length of stay and mortality) and are sustainable (40). These centralized stroke services are configured for thrombolytic therapy and works are underway to examine implementation of ECR (41). It is not clear if this process would result in further reduction in hospitals providing thrombolysis and ECR. Decisions on the number of ECR and thrombolysis capable hospitals serving Sydney would need to be made before deciding on base for MSU.

In summary, any of the ECR-capable hospital can serve as a hub for MSU. We provide a method to identify the hub based on location of suburbs less well-served by other hospital.

## Data Availability

The datasets generated for this study are available on request to the corresponding author.

## Author Contributions

TP: design, analysis, and writing the manuscript. RB, VS, and HM: writing the manuscript.

### Conflict of Interest Statement

TP is on the Advisory Board of Genzyme on Fabry Disease and has received payment for lectures including service on speakers' bureaus for Bayer, Boehringer Ingelheim, Pfizer, and Genzyme. The remaining authors declare that the research was conducted in the absence of any commercial or financial relationships that could be construed as a potential conflict of interest.
